# Characterization of the Wave Phenomenon in Flash-Induced Fluorescence Relaxation and Its Application to Study Cyclic Electron Pathways in Microalgae

**DOI:** 10.3390/ijms23094927

**Published:** 2022-04-28

**Authors:** Priyanka Pradeep Patil, Imre Vass, Milán Szabó

**Affiliations:** 1 Institute of Plant Biology, Biological Research Centre, Eötvös Loránd Research Network, 6726 Szeged, Hungary; patil.priyanka@brc.hu; 2 Climate Change Cluster, University of Technology Sydney, Ultimo, NSW 2007, Australia

**Keywords:** microalgae, photosynthesis, chlorophyll fluorescence, cyclic electron transport

## Abstract

Photosynthesis is a series of redox reactions, in which several electron transport processes operate to provide the energetic balance of light harvesting. In addition to linear electron flow, which ensures the basic functions of photosynthetic productivity and carbon fixation, alternative electron transport pathways operate, such as the cyclic electron flow (CEF), which play a role in the fine tuning of photosynthesis and balancing the ATP/NADPH ratio under stress conditions. In this work, we characterized the electron transport processes in microalgae species that have high relevance in applied research and industry (e.g., *Chlorella sorokiniana*, *Haematococcus pluvialis*, *Dunaliella salina*, *Nannochloropsis* sp.) by using flash-induced fluorescence relaxation kinetics. We found that a wave phenomenon appeared in the fluorescence relaxation profiles of microalgae to different extents; it was remarkable in the red cells of *H. pluvialis*, *D. salina* and *C. sorokiniana*, but it was absent in green cells of *H. pluvialis* and *N. limnetica*. Furthermore, in microalgae, unlike in cyanobacteria, the appearance of the wave required the partial decrease in the activity of Photosystem II, because the relatively high Photosystem II/Photosystem I ratio in microalgae prevented the enhanced oxidation of the plastoquinone pool. The wave phenomenon was shown to be related to the antimycin A-sensitive pathway of CEF in *C. sorokiniana* but not in other species. Therefore, the fluorescence wave phenomenon appears to be a species-specific indicator of the redox reactions of the plastoquinone pool and certain pathways of cyclic electron flow.

## 1. Introduction

### 1.1. Photosynthesis of Microalgae

Microalgae are important resources of valuable metabolites for several biotechnological and industrial applications [1,2,3]. Microalgae, as photosynthetic organisms, convert atmospheric carbon dioxide into biomass using the energy of sunlight. The energy of light is used to extract electrons from water to initiate an electron transfer process, which results in the reduction of NADP^+^ to NADPH. This electron transfer is termed as linear electron flow, and it involves the operation of the two photosystems, Photosystem II (PSII) and Photosystem I (PSI), in series. The electron transport is also regulated by other components such as the cytochrome b_6_/f complex and the mobile plastoquinone (PQ). The photosynthetic electron transport is also coupled to transmembrane proton transport, the energy of which is utilized in ATP synthesis. ATP and NADPH are essential intermediates for the carbon fixation reactions, and these are produced in a defined ratio, which is often insufficient to sustain the carbon fixation processes, especially under stress conditions, in which the ATP demand can increase (reviewed e.g., in [4]). ATP production can be maintained even in the absence of net NADPH production, when the linear electron flow capacity is reduced, e.g., under stress conditions and/or in the absence of CO_2_. This process is termed as cyclic electron flow (CEF), which involves the rerouting the electrons from ferredoxin (Fd) at the acceptor side of PSI to the plastoquinone pool and then back to the vectorial electron transport routes involving cyt b_6_/f and the donor side of PSI, forming thereby a continuous cycling of electrons without transferring it to the carbon fixation reactions (for a recent review see, e.g., [5]). 

### 1.2. Cyclic Electron Flow

Two routes of CEF exist. One of them is the antimycin A-sensitive pathway, which involves the proton gradient regulation 5 (PGR5) and a PGR5-Like Photosynthetic Phenotype 1 (PGRL1) components. The antimycin A-insensitive pathway involves the NAD(P)H dehydrogenase enzymes (NDH) among which two types can be distinguished, the type I and type II NDH (NDH-1 and NDH-2, respectively, reviewed in [6]). In both pathways, electrons are transferred from Fd (or NADPH in the case of NDH-2) to the PQ pool; therefore, these pathways involve pronounced redox changes of the PQ pool and the PSII–PSI intersystem electron transfer chain. The redox changes in the PQ pool have a strong impact on the redox state of the primary quinone acceptor, Q_A,_ which manifests itself in changes in modulated chlorophyll fluorescence levels. 

### 1.3. Flash-Induced Fluorescence Relaxation

A type of Chl fluorescence technique, the flash-induced chlorophyll fluorescence relaxation is an informative tool to monitor electron transfer processes related to the oxidation-reduction events of the PQ pool [7,8]. Illumination with a single turnover flash initiates an electron transfer from the water-oxidizing complex to Q_A_, and the fluorescence yield (or intensity) increases as a result of the formation of Q_A_^−^. The fluorescence intensity then decreases monotonously and is composed of three kinetically well-separated phases of Q_A_^−^ reoxidation (see [7,8]): (i) fast phase, electron transfer to Q_B_ (a mobile electron carrier, which takes up two electrons sequentially from Q_A_) which occurs in 300–500 μs, (ii) middle phase, reoxidation by PQ that binds to the Q_B_ site after the flash, which occurs in 5–15 ms, and (iii) slow phase, which occurs in 10–20 s. The slow phase is due to Q_A_^−^ reoxidation via charge recombination with the oxidized S_2_ (or S_3_) state from the Q_A_Q_B_^−^ state, which is in charge equilibrium with Q_A_^−^Q_B_. The flash-induced fluorescence relaxation under non-stressed or uninhibited conditions is mainly dominated by the fast phase, indicating an efficient electron transfer between Q_A_ and Q_B_ and a largely oxidized PQ pool, and it is quite similar in all photosynthetic organisms. However, modulation of the redox state of the plastoquinone pool results in pronounced changes in the distribution of the three phases as well as the shape of the relaxation curve. 

### 1.4. The Wave Phenomenon of the Fluorescence Relaxation

In the cyanobacterium *Synechocystis* sp. PCC6803, it was observed that under microaerobic conditions, a so-called wave phenomenon appeared, which manifested itself in a dip phase, resulting from the transient oxidation of a strongly reduced PQ pool (because the terminal oxidases oxidizing the PQ pool were not functional due to the low amount of oxygen) and subsequently a fluorescence increase (bump phase), resulting from the re-reduction of PQ pool mediated by the NDH-1 complex [8]. On the other hand, this wave phenomenon could not be observed under microaerobic conditions in green algae [9]. We showed earlier that in *Chlamydomonas reinhardtii*, the combination of the partial decrease in PSII:PSI ratio and microaerobic condition was necessary to induce the wave phenomenon [10]. The characteristics of the flash-induced fluorescence relaxation and the wave phenomenon indicating the oxidation-reduction reactions of the intersystem electron transfer chain are scarcely investigated in microalgae. In this work, we aimed to investigate the wave phenomenon in different microalgae that play an important role in biotechnological and industrial applications: *Chlorella sorokiniana*, which is known as a heat and light-resistant microalga that has high relevance, e.g., in wastewater treatment [11], *Dunaliella salina* and *Haematococcus pluvialis*, which are known as high-content carotenoid producers under stress conditions [12,13,14], and *Nannochloropsis limnetica*, which is a freshwater alga with high total fatty acid content [15]. We investigated the impact of microaerobic environment as well as certain conditions that modulate the relative activity of PSII as compared to PSI on the inducibility of the wave phenomenon in these species. Our aim was to reveal the species and/or condition-specific manifestation of the wave phenomenon and whether it is sensitive to selective inhibitors of cyclic electron flow. 

## 2. Results

### 2.1. Flash-Induced Fluorescence Relaxation under Microaerobic Conditions

First, we screened different microalgae species for the inducibility of the wave phenomenon by applying conditions that were previously shown to induce the wave-type fluorescence relaxation in cyanobacteria. Microaerobic environment is known to cause a strongly reduced plastoquinone pool, since in the absence of their terminal acceptor O_2_, the terminal oxidases are unable to oxidize the reduced PQ molecules [16,17,18], which leads to the activation of fluorescence wave [8]. We found earlier that microaerobic condition alone did not induce the wave in *Chlamydomonas reinhardtii* [9,10]. Therefore, we investigated the effect of microaerobic condition (induced by the glucose oxidase reaction) on the Chl fluorescence relaxation kinetics in various species of microalgae.

**Figure 1:**
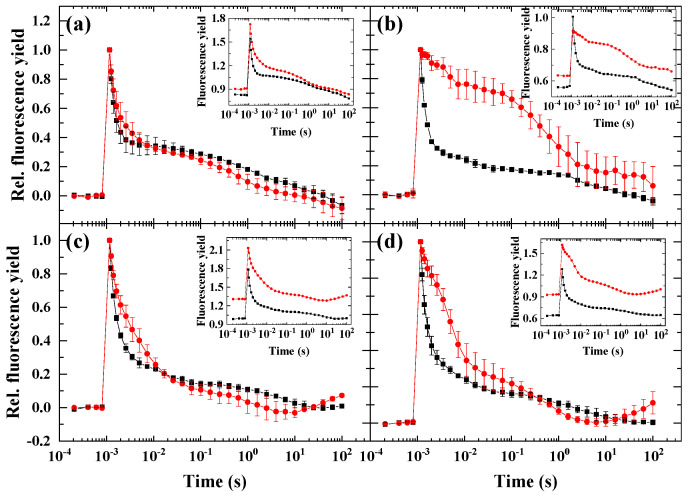
Flash-induced fluorescence relaxation under aerobic (black) and microaerobic (red) conditions. (**a**) *Haematococcus pluvialis* (green cells), (**b**) *Dunaliella salina*, (**c**) *Chlorella sorokiniana*, (**d**) *Nannochloropsis limnetica*. Curves were normalized (F_0_ to 0 and F_m(ST)_, which represents maximal fluorescence after the flash to 1) to achieve the same initial amplitudes. The insets show the original curves, averaged over the three biological replicates, without normalization. Error bars represent the standard deviation of the mean of three biological replicates.

Under microaerobic conditions, all species exhibited an increase in the basic fluorescence, F_0_ (insets of Figure 1), similarly to our earlier observations in *C. reinhardtii* [10]. In agreement with the results obtained earlier in *C. reinhardtii*, the wave phenomenon could not be observed under microaerobic conditions in none of the investigated microalgae species. Interestingly, in *D. salina* (Figure 1b), microaerobic incubation caused a significant slowing down of fluorescence decay and increase in fluorescence both in the fast phase (0–1 ms) and middle phase (10–100 ms), which was followed by a decrease in fluorescence in the slow phase, which is an indication for a reduced PQ pool that slows down forward electron transport from Q_A_^−^ to Q_B_ and enhances the charge recombination of S_2_(Q_A_Q_B_)^−^. In *C. sorokiniana* (Figure 1c) and *N. limnetica* (Figure 1d), a slower fast phase and elevated middle phase could be observed, whereas in *H. pluvialis* (Figure 1a), the decay pattern was not remarkably influenced by microaerobic treatment. 

### 2.2. Flash-Induced Fluorescence Relaxation under Partially Inhibited PSII Activity

One of the crucial factors in the induction of wave phenomenon is the high activity of PSI to oxidize the PQ pool relative to PSII activity, which reduces the PQ pool. Therefore, a condition was applied where PSII activity was largely abolished, but the linear electron flow downstream of Q_A_ toward PSI was undisturbed [10]. 

**Figure 2:**
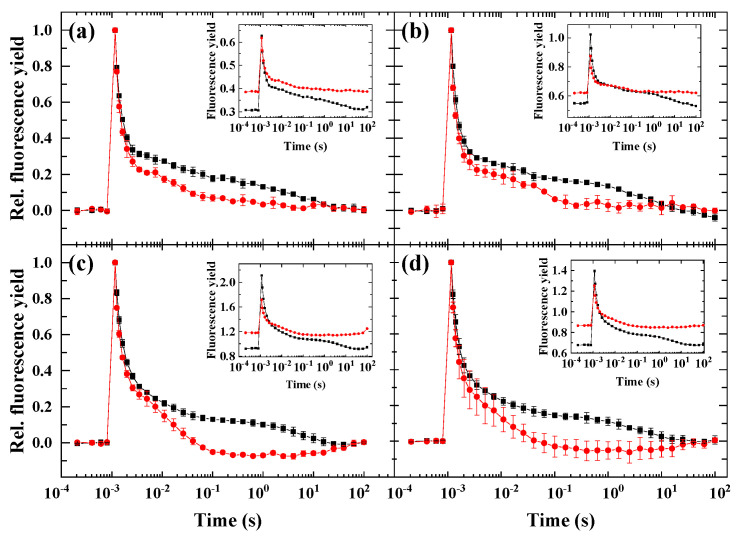
Flash-induced fluorescence relaxation kinetics under aerobic conditions in the absence (black) and in the presence of 1 mM hydroxylamine (red). (**a**) *Haematococcus pluvialis* (green cells), (**b**) *Dunaliella salina*, (**c**) *Chlorella sorokiniana*, (**d**) *Nannochloropsis limnetica*. Curves were normalized (F_0_ to 0 and F_m(ST)_, which represents maximal fluorescence after the flash to 1) to achieve the same initial amplitudes. The insets show the original curves, averaged over the three biological replicates, without normalization. Error bars represent the standard deviation of the mean of three biological replicates.

In the presence of hydroxylamine (HA), the PSII activity was largely abolished, which was indicated by the decreased amplitude of variable fluorescence (F_m_-F_0_) and increased minimal fluorescence (F_0_), but the linear electron flow downstream Q_A_ was undisturbed, as the fast phase of Chl relaxation was similar in non-treated vs. HA-treated cells (Figure 2). In the middle phase of the Chl fluorescence relaxation curve, in the HA-treated cells, the fluorescence was even below the fluorescence level of non-treated cells, indicating the efficient intersystem electron transfer from Q_A_^−^ to Q_B_ and PQ pool. All algae species showed a dip phase in the 1–10 s region, and the lack of slow phase indicates the lack of charge recombination between the Q_B_ and S_2_ state, which was most probably because of the dysfunctional PSII donor side.

### 2.3. Flash-Induced Fluorescence Relaxation under Partially Inhibited PSII Activity Combined with Microaerobic Condition

**Figure 3:**
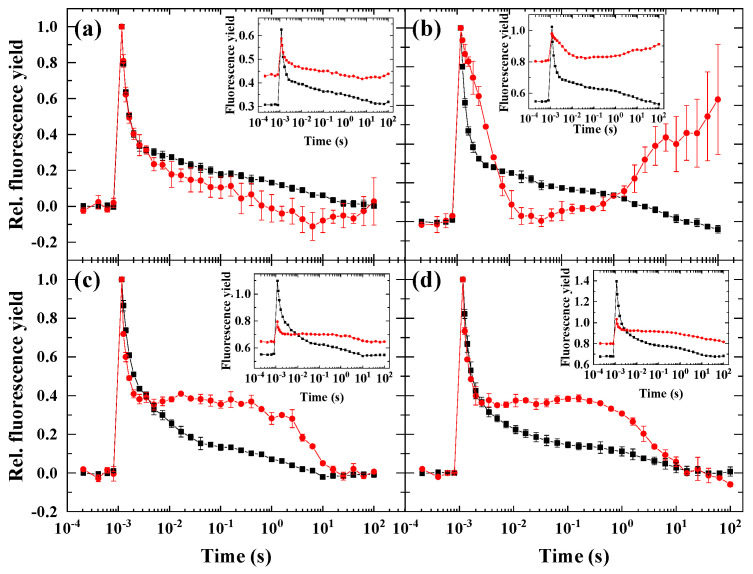
Flash-induced fluorescence relaxation in control aerobic condition (black) and in the presence of 1 mM hydroxylamine (HA) + microaerobic condition (red). (**a**) *Haematococcus pluvialis* (green cells), (**b**) *Dunaliella salina*, (**c**) *Chlorella sorokiniana*, (**d**) *Nannochloropsis limnetica*. Curves were normalized (F_0_ to 0 and F_m(ST)_, which represents maximal fluorescence after the flash to 1) to achieve the same initial amplitudes. The insets show the original curves, averaged over the three biological replicates, without normalization. Error bars represent the standard deviation of the mean of three biological replicates.

When the cells were incubated with HA and then microaerobic treatment was applied, a dip phase was observed in *D. salina* between 0.004 and 1 s (Figure 3b), although the dip was much less expressed than in *C. reinhardtii* [10]. Interestingly, microaerobic condition reversed the fluorescence relaxation characteristics of the HA-treated *C. sorokiniana* and *N. limnetica* cells (Figure 3c,d, respectively, c.f. Figure 2), and an elevated middle phase was observed, but no fluorescence wave appeared. In *H. pluvialis*, the combined HA + microaerobic treatment did not alter significantly the fluorescence relaxation curve (Figure 3a). 

### 2.4. Variability of the Flash-Induced Fluorescence Wave Pattern in the Different Species

Various conditions were found to elicit the wave phenomenon to its largest extent in the different species. It was found earlier that preillumination elicited the wave under aerobic condition in some cyanobacteria [8]; however, this approach has not been applied earlier in microalgae. Therefore, we investigated the effect of preillumination with moderate light, which was applied under the conditions that induced the fluorescence dip phase, which was indicative of the efficient PQ reoxidation by PSI.

**Figure 4:**
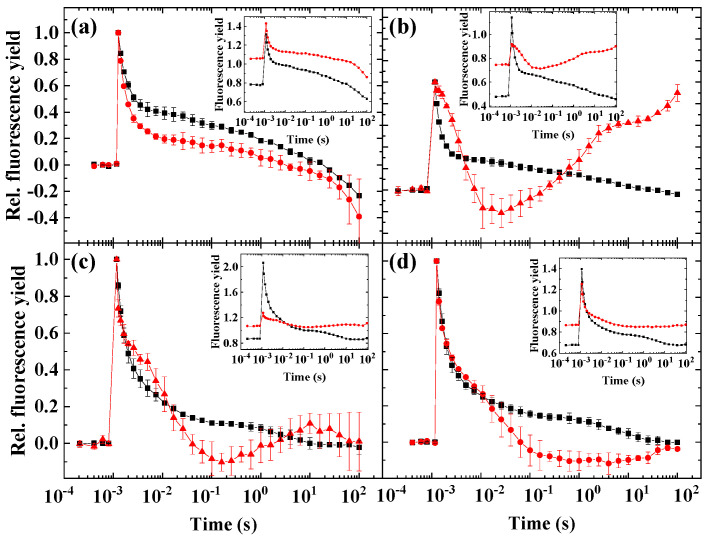
Flash-induced fluorescence relaxation under aerobic control (black) and HA + preillumination (red). (**a**) *Haematococcus pluvialis* (green cells), (**b**) *Dunaliella salina*, (**c**) *Chlorella sorokiniana*, (**d**) *Nannochloropsis limnetica.* The HA + preillumination (red) was applied either under microaerobic condition (**a**,**b**) or under aerobic condition (**c**,**d**). The insets show the original curves, averaged over the three biological replicates, without normalization. Error bars represent the standard deviation of the mean of three biological replicates.

The green cells of *H. pluvialis* did not exhibit any wave feature under the investigated conditions (HA + microaerobic condition, Figure 4a). In *D. salina*, it was observed that the maximum of the dip in fluorescence relaxation occurred at 0.01–0.02 s after preillumination (Figure 4b). As the microaerobic condition in combination with HA was not sufficient to induce the wave phenomenon in *C. sorokiniana* (see Figure 3c), it was investigated under aerobic condition whether preillumination in combination with HA could induce the wave. Under aerobic conditions, after preillumination with moderate light, in the presence of HA, *C. sorokiniana* exhibited the dip at around 0.1–0.2 s, albeit with a relatively low amplitude (Figure 4c). *N. limnetica* did not express the wave phenomenon under the investigated conditions (Figure 4d).

A special condition in the case of *H. pluvialis* is when green cells are transformed to red cells, which accumulate a large amount of astaxanthin. We investigated the fluorescence relaxation phenomenon in red *H. pluvialis* cells as well.

**Figure 5:**
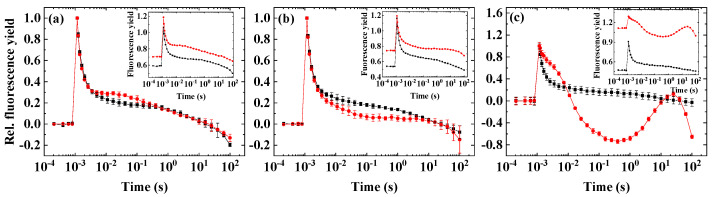
Flash-induced fluorescence relaxation in red cells of *H. pluvialis*. (**a**) Control cells without inhibitor under aerobic (black) and microaerobic (red) conditions. (**b**) HA-treated cells under aerobic conditions (red). (**c**) HA-treated cells under microaerobic conditions (red). The black traces in panels b and c show the aerobic untreated control as in panel a. Curves shown in panels a, b and c were double normalized (F_0_ to 0 and F_m(ST)_, which represents maximal fluorescence after the flash to 1) to achieve the same initial amplitudes. Insets show the original traces, averaged over the three biological replicates, of the respective conditions without normalization. Error bars represent the standard deviation of the mean of three biological replicates.

Red cells of *H. pluvialis* under microaerobic conditions exhibited a slightly elevated F_0_ and middle phase (Figure 5a). HA treatment (Figure 5b) caused similar changes as in other species, i.e., increased F_0_, slight dip in the middle phase and decreased variable fluorescence, indicating that the forward electron flow from Q_A_^−^ to Q_B_ was undisturbed and efficient reoxidation of the PQ pool by PSI occurred. HA + microaerobic treatment induced the fluorescence wave with a large dip at around 0.2–0.6 s, with a subsequent increase in fluorescence (Figure 5c). The characteristics of the wave phenomenon resembled the features observed in *C. reinhardtii* under HA + microaerobic treatment [10], indicating that the partial reduction in PSII activity with a strongly reduced PQ pool favored the formation of the wave in red cells (but not in green cells; see Figure 4a). 

### 2.5. The Effect of Inhibitors of Cyclic Electron Flow on the Fluorescence Wave Phenomenon

It was important to clarify whether the wave phenomenon could be related to cyclic electron flow, mediated either by the NAD(P)H dehydrogenase (NDH-1/2) or the proton gradient regulatory (PGR5/PGRL1) pathways. To this end, various inhibitors specific to these complexes were applied. As *H. pluvialis* green cells and *N. limnetica* did not express the wave, these algae were not examined further.

**Figure 6:**
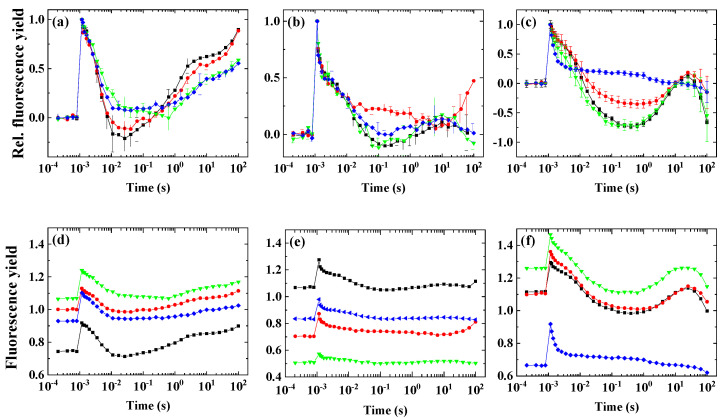
Effect of various inhibitors of cyclic electron flow on flash-induced Chl fluorescence relaxation in *Dunaliella salina* (**a**,**d**), *Chlorella sorokiniana* (**b**,**e**) and *Haematococcus pluvialis* red cells (**c**,**f**). Black squares: samples without additional inhibitor, red circles: treated cells incubated with 4 μM Antimycin A, green triangles: 1.6 mM Polymyxin B, blue triangles: treated cells incubated with 15 μM diphenyleneiodonium (DPI). Panels a, b and c show the double normalized fluorescence traces while panels d, e and f show the original traces without normalization. Error bars represent the standard deviation of the mean of three biological replicates.

In *D. salina*, both polymyxin B and DPI partially blocked the fluorescence wave. Antimycin A was not found to be effective in blocking the wave phenomenon (Figure 6a). In *C. sorokiniana*, the most effective inhibitor was antimycin A, whereas polymyxin B or DPI was not effective (Figure 6b). In red cells of *H. pluvialis*, the wave was partially blocked by antimycin A, it was not blocked by polymyxin B, and it was completely blocked by another NDH-2 inhibitor, DPI (Figure 6c). 

## 3. Discussion

### 3.1. Significance of the Wave Phenomenon in Flash-Induced Chlorophyll Fluorescence Relaxation

Chlorophyll fluorescence kinetics, such as the flash-induced chlorophyll fluorescence relaxation, is a highly informative tool to study the redox regulation of the photosynthetic electron transport. This approach gives valuable information about the efficiency of the sequential electron transfer steps as well as the impact of various stress conditions on the photosynthetic apparatus. Fluorescence relaxation kinetics is therefore a non-intrusive indicator of the photosynthetic responses of particular physiological conditions of microalgae, which encounter variable environmental conditions. As the PQ pool is an important regulatory checkpoint of several interconnecting pathways, chlorophyll fluorescence changes give information not only about the intersystem PSII–PSI electron transfer processes but also about the processes beyond or around PSI, which interconnects with several metabolic pathways.

In addition to its well-distinguishable phases that describe the reoxidation of the reduced Q_A_, special cases of the relaxation patterns could also be identified, which describe the reduction–reoxidation sequences of the plastoquinone pool. This special feature is denoted as the wave phenomenon, which was first observed in the cyanobacteria *Synechocystis* under microaerobic conditions and in *Thermosynechococcus elongatus* under aerobic conditions [8]. This was shown to be related to the operation of NDH-1 in *Synechocystis*. However, in eukaryotic microalgae, the appearance and behavior of the wave phenomenon is far less understood. In *Chlamydomonas*, under microaerobic conditions, the increase in the middle phase of relaxation indicated the higher reduction level of the PQ pool; however, the wave did not appear in this condition, it could only be observed under H_2_-producing conditions, which was found to be related to NDH-2 activity [9]. The appearance of the wave phenomenon is related to the imbalance of the reduction of the PQ pool from PSII electron transport and the reoxidation of PQ by PSI, because if the reoxidation capacity of PSI exceeds the PQ reduction from PSII, a large dip in fluorescence relaxation can be observed. Therefore, as we showed recently, a partial inhibition of PSII activity in relation to PSI activity under microaerobic condition caused an imbalance in the electron injection to and electron withdrawal from the PQ pool and led to the appearance of the wave in *Chlamydomonas reinhardtii* [10]. A similar wave phenomenon could be observed in the red morphotype of the green alga *Acetabularia acetabulum*, indicating a strong reduction of PQ in darkness [19]. However, the wave phenomenon remains largely uncharacterized in eukaryotic algae.

### 3.2. The Role of Impaired PSII Activity and Microaerobic Conditions in the Wave Phenomenon in Microalgae

**Figure 7:**
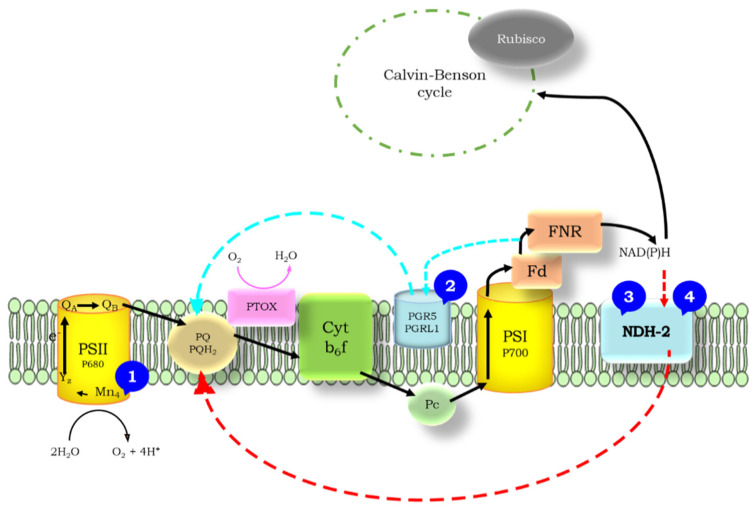
Schematic representation of the electron transfer pathways in microalgae. Specific inhibitors used in this study and sites of their action are represented by blue circles with corresponding numbers: Hydroxylamine (**1**), an inhibitor of the donor side of PSII; Antimycin A (**2**), an inhibitor of the PGR5/PGRL1 pathway, which blocked the wave phenomenon in *C. sorokiniana*; Polymyxin B (**3**); DPI (**4**), inhibitors of the NDH-2 pathway. Polymyxin B and DPI partially blocked the wave in *D. salina*, DPI completely blocked the wave in *H. pluvialis* red cells. The LEF pathway is indicated in black arrows and the NDH-2-dependent and PGR5/PGRL1-dependent CEF pathways taking place inside the membrane are indicated by red and cyan dashed arrows, respectively.

Based on the rationale of our previous work [10], we screened the wave phenomenon in different algae species. Under microaerobic conditions, the stronger reduction state of the PQ pool (indicated by the elevated middle phase of the decay) could be observed in all investigated species—although to different extents, but the wave did not form, which was in accordance with the previous observations in *Chlamydomonas* [9,10,20,21]. This indicates that the wave phenomenon, which could be observed in *Synechocystis* sp. upon microaerobic treatment [8], is either not inducible or it appears with distinct characteristics in eukaryotic microalgae under microaerobic conditions. When the PSII activity was largely diminished by impairing the donor side of PSII by hydroxylamine (Figure 7), which releases the Mn cluster of the water-oxidizing complex [7,22,23], all species behaved similarly, and although the wave was absent, a small dip phase could be observed as a result of the imbalance in electron flow in favor to the reoxidation of PQ pool toward PSI. Under circumstances when both criteria of the induction of wave phenomenon (i.e., the strongly reduced PQ pool in microaerobic condition and the relative decrease in PSII activity to PSI activity) were fulfilled, remarkable differences in the different species could be observed. The most apparent response to the HA + microaerobic treatment could be observed for *D. salina*, as in this species, a wave-like response similar to that of *C. reinhardtii* was recorded, although with smaller amplitude. In *H. pluvialis*, the microaerobic treatment did not affect considerably the behavior of fluorescence relaxation of HA-treated cells when the PSII activity was partially inhibited, indicating that the decreased PSII activity in combination with highly reduced PQ pool was still not sufficient to elicit the wave phenomenon in this species (in green cells). In *C. sorokiniana* and *N. limnetica*, the microaerobic treatment completely eliminated the fluorescence decrease in HA-treated cells, indicating that the formation of the wave is probably dependent on O_2_-consuming pathways such as chlororespiration [24]. 

### 3.3. Different Conditions Are Required to Induce the Wave Phenomenon in Different Species

Therefore, it appears that the ‘wave-inducing’ condition that was established for *C. reinhardtii* is not generally applicable for microalgae, and the different species rely on different pathways and mechanisms to induce the wave. We found that preillumination was not essential to induce the wave in *D. salina*, but it enhanced its formation, as indicated by a larger dip phase as compared to dark-acclimated cells. In *C. sorokiniana*, preillumination was required for the induction of a wave in HA-treated cells, whereas preillumination was ineffective in the modulation of fluorescence relaxation in *N. limnetica* and *H. pluvialis*. However, a special case has to be considered in *H. pluvialis*. As these algae are able to produce a large amount of astaxanthin under stress conditions, they also turn into red cells (haematocysts) from green (or vegetative) cells with concomitant remodeling of several metabolic reactions, photosynthetic activity and photoprotective capability (e.g., [25,26,27,28,29]). Red cells exhibited enhanced cyclic electron flow [27,28]; in agreement with this observation, we found that red cells exhibit a large wave phenomenon as compared to green cells when treated with HA and microaerobic condition. On the other hand, other studies found that both PSI and PSII activity declined under light stress, which could be related to the loss of cytochrome b_6_/f in red cells and consequently the decreased linear and cyclic electron transport [30,31]. Nevertheless, significant activity of the photosystems remained in red cells, and from the flash-induced fluorescence analysis of green cells vs. red cells, it appears that the PQ-oxidizing capacity of PSI relative to the PQ-reducing capacity from PSII is larger in red cells than in green cells, as demonstrated by the existence of the wave phenomenon in red cells but not in green cells (c.f. Figure 4a and Figure 5c). When comparing *D. salina* and *H. pluvialis*, it was found that the CEF capacity is larger in the former species under high light stress [31]. This is in agreement with our observation that whereas *D. salina* exhibited a clear wave phenomenon under HA + microaerobic condition (Figure 3b), green cells of *H. pluvialis* did not express the wave (Figure 3a). Red *H. pluvialis* cells exhibited the wave, but the dip occurred much more slowly (although with larger amplitude) as compared to *D. salina* (c.f. Figure 3b and Figure 5c). The relation of cyclic electron flow to the wave phenomenon in the algae species applied in this study therefore requires further investigation.

### 3.4. Impact of Inhibitors of Cyclic Electron Flow on the Fluorescence Wave Phenomenon in Microalgae

Cyclic electron flow around PSI has been shown to operate in algae species that are investigated here [32,33,34,35,36] and reviewed also in [37]; however, its mechanisms remain largely uncharacterized in microalgae. Cyclic electron flow is regulated by the PGR5/PGRL1 pathway and/or the NDH-1/2 pathway, although recent findings suggest that the role of these complexes in mediating fast CEF is indirect [5,38]. In *Chlamydomonas*, the NDH-2 complex plays a role in the reduction of PQ pool that elicits the wave formation, and it was found that polymyxin B is an efficient inhibitor of the wave phenomenon in this species [9,10]. The PGR5 pathway, although operational in *Chlamydomonas*, was not found to be contributing to the wave formation, as it was largely insensitive to antimycin treatment [9,10]. In the current study, the situation is the opposite for *Chlorella sorokiniana*, as antimycin efficiently blocked the wave, whereas polymyxin was ineffective; therefore, it seems that the antimycin-sensitive PGR5 pathway is an essential component of the wave formation and of CEF in this species (Figure 7). 

However, antimycin A was less effective in the red cells of *H. pluvialis* and *D. salina*, indicating that the role of the antimycin-sensitive pathway in the formation of the wave is less pronounced as compared to *C. sorokiniana*. Regarding the role of the NDH-2 pathway in these species, this pathway probably does not operate in *C. sorokiniana*, or at least, it does not contribute to the formation of the fluorescence wave. In other species, this remains to be clarified, because the inhibitor polymyxin B had no inhibitory effect, whereas DPI fully blocked the wave formation in *H. pluvialis* red cells (Figure 7). DPI blocked the NADPH-induced non-photochemical reduction of the PQ pool in *C. reinhardtii*, indicating the involvement of NDH-2 in this process [39]. The complete inhibition of the wave formation by DPI in red cells of *H. pluvialis* seems to suggest the involvement of the NDH-2 pathway in this species as well; however, the operation of the NDH-2 pathway and its involvement in cyclic electron flow remains to be further clarified. Nonetheless, we can conclude that the antimycin-sensitive PQ pool reduction plays a crucial role in the wave formation in *Chlorella sorokiniana*, whereas this pathway seems less significant in other microalgae (Figure 7). 

## 4. Materials and Methods

### 4.1. Algal Cultures

Algal cultures were obtained from the Culture Collection of Autotrophic Organisms (CCALA), Centre for Phycology, Institute of Botany of the AS CR, Trebon, Czech Republic. *Chlorella sorokiniana* (Trebouxiophyceae) and *Nannochloropsis limnetica* (Eustigmatophyceae) were grown in BG-11 medium with the light intensity of 56 μmol photons m^−2^ s^−1^ white light, 12 h: 12 h day: night diurnal cycle with continuous shaking at 120 rpm, at a constant temperature of 24 °C. *Dunaliella salina* (Chlorophyceae) was grown under the same conditions as described above but in f/2 medium (prepared with artificial seawater) supplemented with 30 gL^−1^ NaCl and *Haematococcus pluvialis* (Chlorophyceae) was grown in BG11 medium at 100 μmol photons m^−2^ s^−1^ white light without shaking. For measurements, the cells were harvested in the early exponential growth phase (OD at 720 nm of 0.2–0.3) and approx. 1 h after the onset of the daily light cycle, centrifuged at 6500× *g* for 5 min at 24 °C and were resuspended in fresh medium to adjust the Chl *a* content of 5 μg mL^−1^. Chl content was determined using UV-visible spectrophotometer by acetone:DMSO extraction according to [40]. For the *Haematococcus* red cyst cells, the green cells were taken from the exponential phase (OD at 720 nm of 0.3–0.4). The cells were centrifuged at 6000× *g* for 5 min at 24 °C and suspended in fresh nitrate-free BG11 medium including 50 mM sodium acetate, illuminated with the light intensity of 100 μmol photons m^−2^ s^−1^ for 7 days. The culture was prevented from shaking to avoid aggregation, and the flask was gently swirled every two days. On the day of measurement, the red cyst cells were used without resuspending into the fresh medium. 

### 4.2. Experimental Procedure

The initial control (aerobic condition) was measured after 3 min of the dark adaptation; in the same sample, microaerobic condition (O_2_ content of <1 µmol L^−1^) was achieved by the addition of 10 mM glucose, 7 U mL^−1^ glucose oxidase and 60 U mL^−1^ catalase. Then, it was incubated in the dark for 15 min, after which the fluorescence decay curves were measured in the microaerobic state. To test the hydroxylamine effect (NH_2_OH, abbreviated as ‘HA’ thereafter), after measuring the aerobic control, the samples were further incubated for 3 min in the dark with 1 mM HA, and fluorescence curves were recorded. Subsequently, the same samples were subjected to microaerobic conditions in the presence of HA, and fluorescence decay curves were measured (mentioned as ‘HA + microaerobic conditions’). For the appearance of a wave, in *Haematococcus*, red cells were taken from the same growth flask without washing. The aerobic control was measured after 3 min in the dark, further incubating the cells for 10 min in the dark with the addition of 3 mM HA followed by the similar procedure of HA + microaerobic condition. *Chlorella sorokiniana* cells were preilluminated for 1 h with 100 μmol photons m^−2^ s^−1^ white LED light, which was followed by 3 min in the dark to measure aerobic control. The cells were then incubated for 2 min in the dark with 1 mM HA, and the fluorescence curves were recorded. In the case of *Dunaliella salina*, the cells were preilluminated for 1:30 h with 100 μmol photons m^−2^s^−1^ white LED light followed by the similar procedure of HA + microaerobic conditions. For the treatments with inhibitors, the above procedure was applied in the presence of 4 μM Antimycin A, 1.6 mM Polymyxin B and 15 μM Diphenyleneiodonium (DPI). These inhibitors were added to the cell suspensions in the dark with 3 min incubation before the application of HA and microaerobic treatment. Experiments were performed in triplicates, and averaged fluorescence traces with standard deviation were plotted using OriginPro (OriginLab Corp., Northampton, MA, USA).

### 4.3. Flash-Induced Chlorophyll Fluorescence Relaxation Kinetics

Flash-induced chlorophyll fluorescence yield was measured using a double-modulation fluorometer (FL-3000, Photon System Instruments, Brno, Czech Republic) [41] as described in [10]. A 2 mL sample was placed in a cuvette with 1 cm pathlength and was continuously stirred with a small magnetic stirrer bar in the dark. Four measuring flashes (8 μs, separated with 200 μs intervals, wavelength of 620 nm) were applied to determine minimum fluorescence in the dark (F_0_), after which a single turnover saturating actinic flash (30 μs, wavelength of 639 nm) was given to induce the formation of Q_A_^−^, which resulted in the rise of fluorescence intensity (denoted as F_m(ST)_). The fluorescence decay resulting from the reoxidation of Q_A_^−^ was measured by applying measuring flashes in the time range from 150 µs to 100 s on a logarithmic time scale. 

## 5. Conclusions

Flash-induced Chl fluorescence relaxation is a valuable non-intrusive tool to investigate several interconnecting electron transport pathways, and it has a useful potential for detecting alterations in the photosynthetic activity in microalgae that have high relevance in biotechnology and applied research in broader scales. A specific and informative form of Chl fluorescence relaxation is the so-called wave phenomenon, which has been investigated in cyanobacteria [8] and some model species of green algae, such as *Chlamydomonas reinhardtii* [9,10]. In the present study, we found that the conditions that lead to the formation of the wave phenomenon are different in different microalgae and seem to be species-specific.

According to previous results, the two critical conditions for the fluorescence wave were the highly reduced PQ pool under microaerobic condition and the lower capacity of PSII to reduce the PQ pool as compared to the oxidizing capacity of PSI [10]. The earlier results have also pointed to the essential role of the NDH complexes (NDH-1 in cyanobacteria [8] and NDH-2 in *Chlamydomonas* [9,10]) in mediating the CEF process, which is reflected by the fluorescence wave. We could demonstrate here that combination of an increased level of PQ pool reduction and limited PSII activity was sufficient to induce the fluorescence wave in red cells of *H. pluvialis* and in *D. salina*. However, other species either did not show the wave (*H. pluvialis* green cells and *N. limnetica*), or it appeared under different conditions (*C. sorokiniana*, under preillumination + HA treatment in aerobic condition). Although our results indicate the involvement of the PGR5/PGRL1 pathway in *C. sorokiniana* and the NDH-2 pathway in *H. pluvialis* red cells, the wave formation could not be assigned unequivocally to cyclic electron flow, partly because the kinetics of the PQ reoxidation and re-reduction occurs markedly slower that the rapid turnover of CEF. Certainly, other processes such as mitochondrial respiration and chlororespiration could potentially donate electrons to the PQ pool and therefore modulate the wave phenomenon; these processes do not operate under microaerobic condition. However, the chlororespiration likely contributes to wave formation in *C. sorokiniana*, because microaerobic treatment blocked the wave formation unlike in other species, and preillumination under aerobic condition (which also might enhance chlororespiration) enhanced the wave. Therefore, multiple routes in the different microalga species might contribute to the wave formation, which need further investigation.

## Data Availability

Not applicable.
